# Characterization of the Complete Mitochondrial Genome of *Castanopsis tibetana* Hance: A Precious Timber Species

**DOI:** 10.3390/genes17040430

**Published:** 2026-04-07

**Authors:** Zi-Fei Wang, Zhi-Tong Xiao, Xiao-Long Jiang, Feng Song, Fei Liu

**Affiliations:** 1 School of Forestry, Central South University of Forestry and Technology, Changsha 410004, China; 18692271142@163.com (Z.-F.W.); 19313072315@163.com (Z.-T.X.); xiaolongjiang1@gmail.com (X.-L.J.); 2 Guangxi Forestry Research Institute, Nanning 530002, China

**Keywords:** *Castanopsis tibetana*, mitochondrial genome, repeated sequences, genome recombination, phylogenetic relationship

## Abstract

Background/Objectives: *Castanopsis tibetana* Hance (*C. tibetana*) is a valuable timber species in southern China. Its chloroplast and nuclear genomes have been characterized, but its mitochondrial genome (mitogenome) remains unknown. This study assembles and characterizes the first complete mitogenome of *C. tibetana*, elucidating its structural and evolutionary features. Methods: A hybrid approach combining Oxford Nanopore long reads and Illumina short reads was used. The mitogenome was assembled via iterative seed-based mapping and annotated via GeSeq and tRNAscan-SE. Repeats were identified via MISA, TRF, and REPuter. The RNA editing sites were predicted with the PREP suite. Phylogenetic analysis was performed on 14 conserved protein-coding genes from 13 species via maximum likelihood and Bayesian inference. Results: The mitogenome is a 554,078 bp circular molecule (GC 45.27%) encoding 51 genes (32 PCGs, 16 tRNAs, 3 rRNAs). It contains 202 simple sequence repeats (37.1% tetrameric). We predicted 53 C-to-U RNA editing sites, most frequently in *nad7* and *nad5*. Codon usage showed bias, with 28 codons having RSCU > 1. Twenty fragments (6001 bp, 1.08% of the mitogenome) were transferred from the chloroplast. Phylogenomic analysis placed *C. tibetana* within Fagaceae, close to other *Castanopsis* species. Conclusions: This study provides the first comprehensive characterization of the *C. tibetana* mitogenome, revealing its structural architecture, repetitive landscape, RNA editing profile, and phylogenetic placement. These findings offer valuable genomic resources for understanding mitogenome evolution in Fagaceae and support future research on the conservation genetics and molecular breeding of this important tree species.

## 1. Introduction

Fagaceae, the beech–oak family, comprises over 900 species across eight genera within Fagales [1,2]. Its members are predominantly distributed in temperate, subtropical, and tropical forests across the Northern Hemisphere [3], although fossil evidence—including a 52 Ma infructescence of *Castanopsis* and associated leaves from the Laguna del Hunco flora in southern Argentina—extends its historical range to the Southern Hemisphere during the early Eocene [4]. The family plays a critical ecological role, particularly in Southeast Asia and South Asia, where species-rich genera such as *Quercus*, *Castanea*, and *Castanopsis* contribute to forest ecosystem stability and biodiversity maintenance [5,6]. Economically, Fagaceae provides valuable timber and non-timber products, including starch-rich nuts from *Castanopsis* species.

Recent genomic efforts have focused predominantly on *Quercus*, with over ten assembled genomes [7,8]. In contrast, genomic resources for *Castanopsis* remain limited. The first *Castanopsis* genome, that of *Castanopsis tibetana (C. tibetana),* was published in 2021, revealing a size of 878.6 Mb and a repetitive sequence content of 54.3% [9]. The genomes of *Castanopsis hystrix* and *Castanopsis carlesii* (*C. carlesii*) were subsequently reported, revealing significant expansion and contraction of gene families potentially linked to adaptation to tropical-subtropical climates [10]. Moreover, high-throughput sequencing has greatly advanced chloroplast genomics in Fagaceae, refining phylogenetic and evolutionary inference [11,12]. However, investigations into mitochondrial genomes (mitogenomes) within the family remain scarce, with only a handful of species characterized to date [8,13]. This disparity has resulted in a critical gap in our understanding of the structural evolution, recombination dynamics, and phylogenetic utility of mitogenomes in this ecologically and economically important lineage.

Among Fagaceae, *Castanopsis* represents a genus of approximately 120 evergreen tree species that dominate subtropical broadleaf forests in East Asia and Southeast Asia [14,15]. The genus is thought to have diverged from its ancestor approximately 52 million years ago [4], with most extant species arising from rapid diversification during the Neogene [16,17]. Unlike many temperate lineages that experienced range shifts during Quaternary glaciation cycles [14,18], *Castanopsis* species exhibit relatively stable historical distributions [19,20]. This stability, combined with outcrossing reproductive systems and minimal domestication [14], makes *Castanopsis* an ideal system for investigating how natural evolutionary processes shape genomic variation over long timescales.

*C. tibetana*, an endemic species widely distributed in humid subtropical regions south of the Yangtze River to eastern Yunnan, typically below 1500 m in elevation [21], is a keystone component of these ecosystems [9]. Morphologically, it is characterized by grayish-brown fissured bark, coriaceous ovate-elliptic leaves with serrate margins in the upper half, spiny cupules, and conical nuts covered with brown villous hairs. Ecologically, it provides habitat and food for diverse fauna, contributes to soil and water conservation, and enhances forest resilience. Its dense, durable timber is highly valued for construction and furniture, supporting local economies [21]. Despite its ecological and economic importance, research on *C. tibetana* has focused mainly on morphology, ecophysiology, population genetics, and chloroplast genomics [9], leaving its mitogenome entirely unexplored.

Compared with their animal counterparts, plant mitogenomes are markedly more complex. They exhibit large and variable sizes, multipartite structures, dynamic rearrangements, and fluid gene content shaped by frequent recombination, intracellular gene transfer, and horizontal gene acquisition [22]. These genomes are rich in repetitive sequences, introns, and non-coding DNA and offer valuable models for studying genome evolution and structural plasticity [23,24]. Although core gene sets are highly conserved and evolve slowly—providing useful markers for deep phylogenetics—structural heterogeneity and complexity pose substantial challenges for assembly. Consequently, long-read sequencing technologies have become essential for obtaining complete and accurate mitogenome assemblies [25,26].

To address the knowledge gap in Fagaceae mitogenomes and explore their phylogenetic potential, we assembled and annotated the first complete mitogenome of *C. tibetana* via a hybrid approach combining Oxford Nanopore long reads and Illumina short reads. We characterized its structural architecture, repetitive landscape, recombination events, codon usage bias, cross-organellar gene transfer, and RNA editing sites. Furthermore, we performed comparative phylogenetic analyses to resolve its placement within Fagaceae [27,28]. This study aims to (i) characterize the structural and sequence features of the *C. tibetana* mitogenome, with a focus on repeat-mediated structural dynamics; (ii) assess its phylogenetic position within Fagaceae via a robust comparative framework; and (iii) establish high-quality genomic resources to support future research on population genetics, conservation, and sustainable management of this important tree species [9,29]. By providing the first insights into its mitogenome, this study lays a foundation for understanding the biogeography and evolutionary history of *C. tibetana* and informs conservation strategies in the context of environmental change [30].

## 2. Materials and Methods

### 2.1. Sequencing Data Retrieval

The raw Nanopore data (CNX0301373) and the raw Illumina data of the genome (CNX0301368) were retrieved from the CNGB Sequence Archive (CNSA) of the China National GenBank DataBase (CNGBdb). The data are publicly available under the CNGBdb project number CNP0001714 [9].

### 2.2. Genome Assembly and Annotation

The mitogenome of *C. tibetana* was assembled via both de novo and homology-based approaches [31]. A seed-based iterative mapping approach was used to extract mitochondrial reads from the Nanopore dataset. Briefly, a BLAST+ v2.12.0+ [32,33] database was constructed from preliminary assemblies generated with SMARTdenovo v5.34.0 [34] and Canu v2.3 [35]. This database was queried against mitogenomes of closely related species to identify seed sequences. Minimap2 v2.22 [36,37] was then used to map raw Nanopore reads to these seeds (parameters: overlap > 1 kb, similarity > 70%). Reads meeting these criteria were added to the seed set, and the process was repeated until no further mitochondrial reads could be identified. The extracted reads were assembled by Flye v2.8.1 [38,39], and the resulting graph was visualized with Bandage v0.8.1 [40] to resolve circular structures. Iterative error correction of the mitochondrial sequences was performed via Minimap2 and Racon v1.4.20 [41].

For homology-based validation, GetOrganelle v1.7.5 [42] was used to assemble mitochondrial reads from Illumina data using related species as references. Candidate sequences from both de novo and homology-based approaches were assembled with Unicycler v0.4.8 [43] and SPAdes v3.15.5 [44]. *Arabidopsis thaliana* (L.) Heynh. served as the reference genome for protein-coding genes (PCGs). The final circularized mitogenome was annotated via GeSeq v2.03 [45], with tRNA genes validated by tRNAscan-SE v2.0.7 [46] and rRNA genes by BLASTN. Manual curation was performed via Geneious Prime v2025.0.2. The final annotated mitogenome was deposited in GenBank [47].

### 2.3. Analysis of Rscu and Repeated Sequences

The protein-coding sequences of the genome were extracted via Phylosuite v1.2.3 [48]. Mega [49] was used to analyze mitochondrial PCG codon usage and calculate RSCU values. MISA [50] (https://webblast.ipk-gatersleben.de/misa/, accessed on 3 January 2025) employs the parameters “1-10 2-5 3-4 4-3 5-3 6-3”, TRF [51] (https://tandem.bu.edu/trf/trf.unix.help.html, accessed on 4 January 2025), and REPuter web server [52] (https://bibiserv.cebitec.uni-bielefeld.de/reputer/, accessed on 4 January 2025) to identify repeated sequences, including simple sequence repeats (SSRs), tandem repeats, and interspersed repeats. The results were visualized via TBtools-II v2.154 [53].

### 2.4. Detection of Genome Recombination

To assess recombination potential, all 202 repetitive sequences identified via BLASTN were extracted along with their 1000 bp flanking regions. A simulated “recombined” conformation was generated by exchanging the flanking sequences. The long-read data were mapped to both the primary and simulated conformations to evaluate the potential for recombination events.

### 2.5. Chloroplast to Mitochondrion DNA Transformation and RNA Editing Prediction

The mitochondrial and chloroplast sequences were compared via BLASTN. Since the chloroplast genome has a duplicated region, to avoid double-counting, we removed alignments from the duplicated region and extracted the MtPts sequence for annotation to identify tRNAs and chloroplast-encoded gene fragments transferred from the chloroplast sequence. Finally, we used TBtools-II v2.154 for visualization. The prediction of RNA editing events was based on the PREP suite website [54] (http://www.prepact.de/prepact-main.php, accessed on 5 January 2025).

### 2.6. Synteny and Phylogenetic Analysis

Based on sequence similarity determined by BLASTN, the synteny between *C. tibetana* and its closely related species, *C. carlesii* (PP853255.1) and *Lithocarpus litseifolius* (NC_065018.1) (*L. litseifolius*), was analyzed and visualized via LINKVIEW2 (https://yangjianshun.github.io/LINKVIEW2/, accessed on 6 January 2025). The mitogenomes of closely related species were selected and downloaded from NCBI (https://www.ncbi.nlm.nih.gov/, accessed on 21 January 2026) on the basis of their affinity, and then PhyloSuite v1.2.3 was used to extract shared genes. MAFFT v7.313 [55] with a bootstrap value of 1000 was used for multiple sequence alignment analysis. IQ-TREE v1.6.8 [56] and MRBAYES v3.2.6 [57] were used for phylogenetic analysis. The results of the phylogenetic analysis were visualized via ITOL v7 software.

## 3. Results

### 3.1. Morphological Features of C. tibetana 

**Figure 1:**
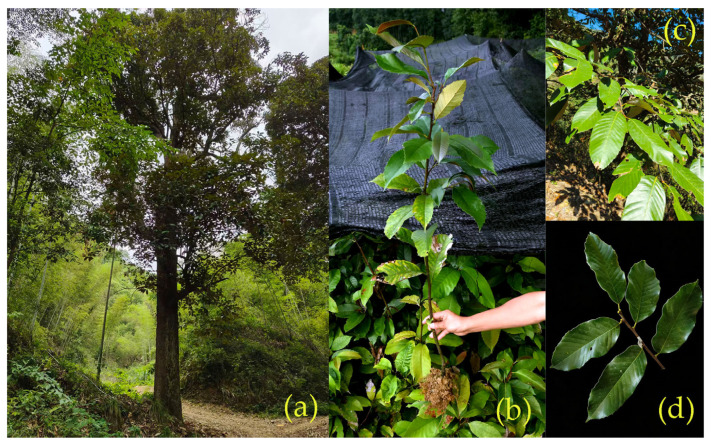
Morphological features of *Castanopsis tibetana* (*C. tibetana*). (**a**) Mature tree in its natural forest habitat. (**b**) Juvenile individual. (**c**) Branches and foliage. (**d**) Close-up view of leaves.

*C. tibetana* is an evergreen tree species that can reach heights of 20–25 m and is characterized by a broad crown and grayish-brown bark that becomes deeply fissured with age (Figure 1a). Juvenile individuals exhibit a more compact growth form than mature trees do (Figure 1b). The leaves are coriaceous and ovate-elliptic in shape, with serrate margins confined to the upper half (Figure 1c,d).

### 3.2. General Features of the C. tibetana Mitogenome

**Figure 2:**
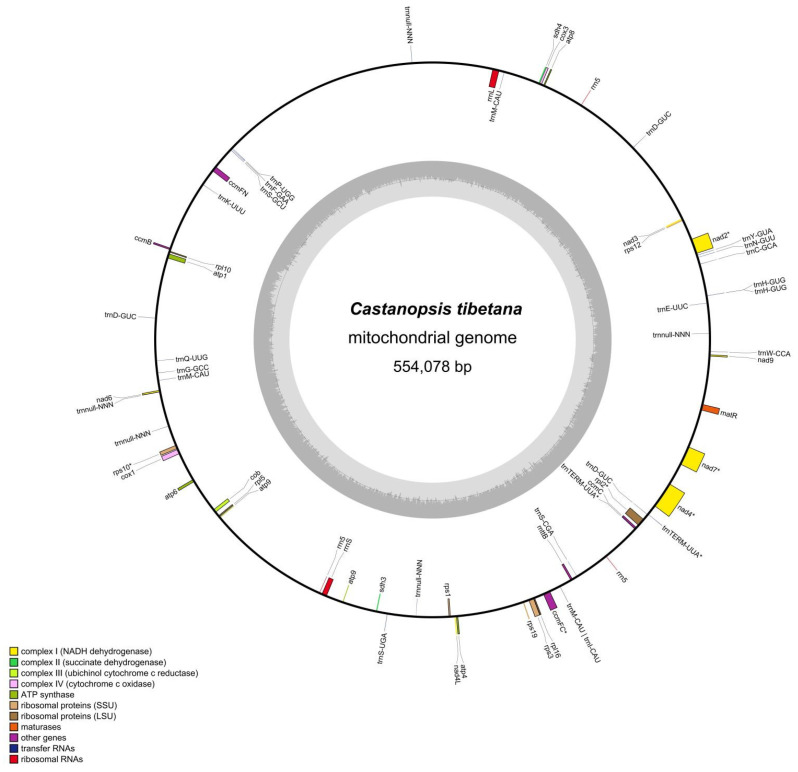
Gene map of the *C. tibetana* mitochondrial genome. Genes are displayed on the outer and inner sides of the circle. Asterisks (*) represent intron-containing genes. The dark gray areas in the inner circle represent the GC content.

The mitogenome of *C. tibetana* was assembled as a circular molecule of 554,078 bp, with a GC content of 45.27% (Figure 2). Coverage depth analysis confirmed the high continuity of the assembly and the absence of large-scale assembly errors, with base-level coverage profiles showing no significant coverage drops or extreme outliers. These data further support that the circular conformation was inferred computationally and validated by long-read sequencing (Figure S1). Annotation revealed a total of 51 genes, comprising 32 protein-coding genes (PCGs), 16 transfer RNA (tRNA) genes, and three ribosomal RNA (rRNA) genes (Figure 2; Table S1). The PCGs included 21 core genes involved in oxidative phosphorylation and 11 non-core genes encoding ribosomal proteins (large subunits: *rpl2*, *rpl5*, *rpl10*, and *rpl16*; small subunits: *rps1*, *rps3*, *rps10*, *rps12*, and *rps19*) and succinate dehydrogenase subunits (*sdh3* and *sdh4*). The core genes included five ATP synthase genes (*atp1*, *atp4*, *atp6*, *atp8*, and *atp9*), seven NADH dehydrogenase genes (*nad2*, *nad3*, *nad4*, *nad4L*, *nad6*, *nad7*, and *nad9*), four cytochrome c biogenesis genes (*ccmB*, *ccmC*, *ccmFC*, and *ccmFN*), two cytochrome c oxidase genes (*cox1* and *cox3*), one apocytochrome b gene (*cob*), one maturase gene (*matR*), and one transport membrane protein-encoding gene (*mttB*). Notably, *nad7* was retained in the mitogenome, indicating that no functional transfer to the nucleus occurred in this species.

### 3.3. PCGs Codon Usage Analysis

**Figure 3:**
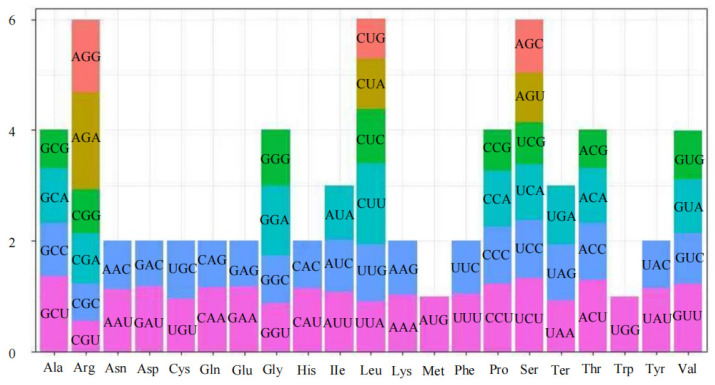
Relative Synonymous Codon Usage (RSCU) in the *C. tibetana* mitochondrial genome. Codon families are displayed on the x-axis. The RSCU values represent the observed frequency of a specific codon relative to the expected frequency of that codon under uniform synonymous codon usage.

Codon usage analysis of the 32 PCGs revealed patterns of synonymous codon preference. Relative synonymous codon usage (RSCU) values indicated that 28 codons were used more frequently than expected (RSCU > 1), with GCU (Ala), UCU (Ser), and ACU (Thr) being the three most prevalent codons (Figure 3). As expected, the start codon AUG (Met) and tryptophan codon UGG (Trp) presented RSCU values of 1, reflecting the absence of synonymous alternatives. This bias is consistent with patterns observed in other Fagaceae mitogenomes, suggesting conserved translational constraints.

### 3.4. Repetitive Sequence Characterization

**Figure 4:**
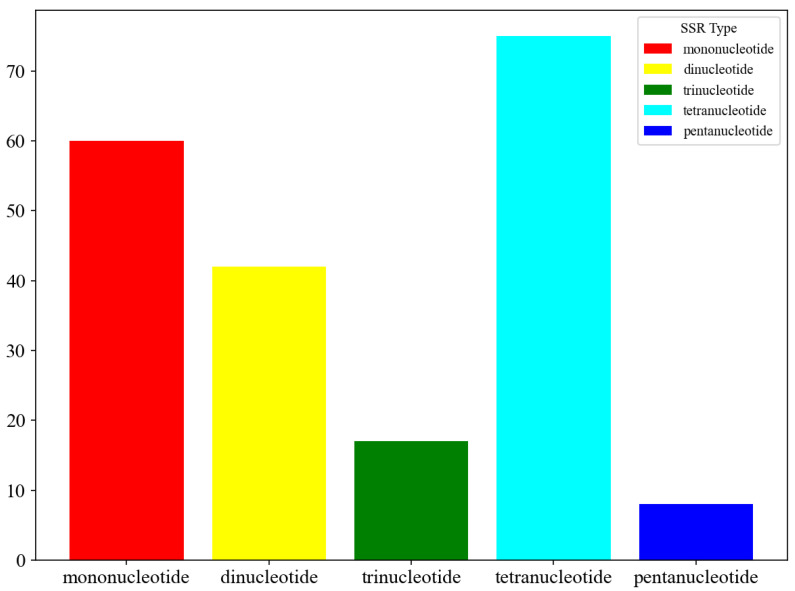
SSR type distribution for *C. tibetana*.

A total of 202 simple sequence repeats (SSRs) were identified in the *C. tibetana* mitogenome. Tetrameric repeats were the most abundant class (*n* = 75; 37.1%), followed by mononucleotide (*n* = 60; 29.7%), dinucleotide (*n* = 42; 20.8%), and trinucleotide repeats (*n* = 17; 8.4%). No hexanucleotide SSRs were detected (Figure 4 and Table S1).

### 3.5. Chloroplast to Mitochondrion DNA Transformation

**Figure 5:**
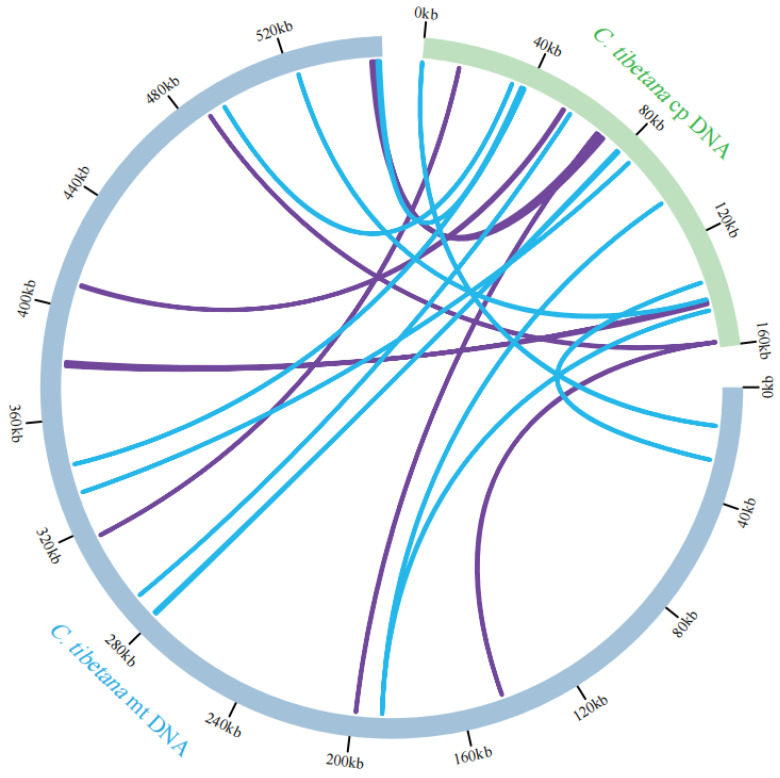
Schematic diagram of gene transfer between the chloroplast and mitochondrial genomes of *C. tibetana*. The blue and green arcs represent the mitochondrial and chloroplast genomes, respectively, and the lines connect the corresponding parts between the homologous regions of the mitochondrial and chloroplast genomes. The purple lines indicate a similarity of 70% to 90% between the sequences, while the blue lines represent the contents with a similarity higher than 90%.

Sequence similarity searches against the *C. tibetana* chloroplast genome (NC_065324.1) revealed 20 homologous fragments putatively transferred to the mitochondrion, with a combined length of 6001 bp. These mitochondrial plastid DNA (MTPT) sequences constitute 1.08% of the total mitogenome length (Figure 5, Table S2).

### 3.6. The Prediction of RNA Editing

**Figure 6:**
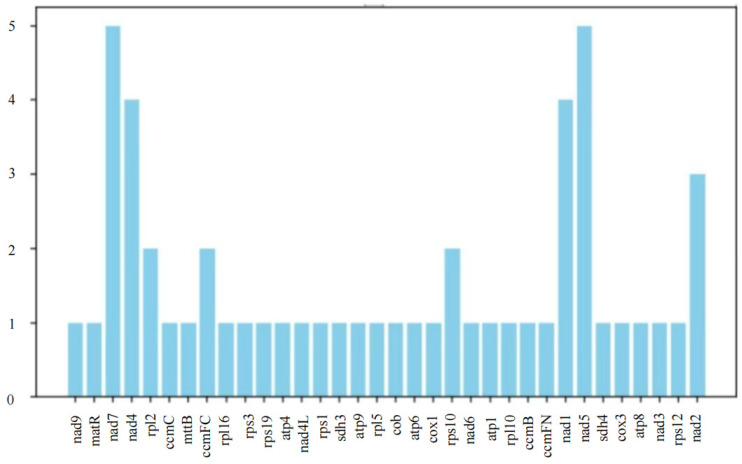
Number of RNA editing sites predicted by individual PCGs in mitochondria.

Using the PREP suite with a cutoff value of 0.2, 53 potential C-to-U RNA editing sites were predicted across the 32 mitochondrial PCGs (Figure 6). The greatest number of editing sites was found in nad7 and nad5 (five sites each), followed by nad4 and nad1 (four sites each). No U-to-C edits were detected.

### 3.7. Synteny and Phylogenetic Analysis

**Figure 7:**
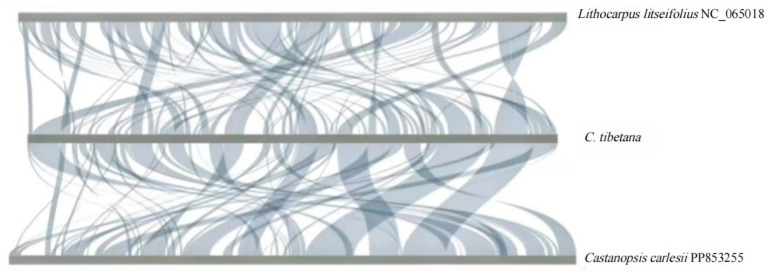
Mitochondrial Genome Homology. The bar chart represents the mitochondrial genome, whereas the ribbon diagram shows the homologous sequences between adjacent species. The gray areas indicate regions of good homology. Common blocks shorter than 0.5 kb are not retained, and regions without common blocks suggest that they are species-specific. *C. carlesii* (PP853255.1) and *L. litseifolius* (NC_065018.1) were compared with *C. tibetana*.

Comparative synteny analysis of three Fagaceae mitogenomes—*C. tibetana*, *C. carlesii*, and *L. litseifolius*—revealed extensive structural rearrangements. While several homologous collinear blocks were identified, many were short and fragmented, indicating limited conservation of mitogenome architecture within the genus *Castanopsis* (Figure 7).

**Figure 8:**
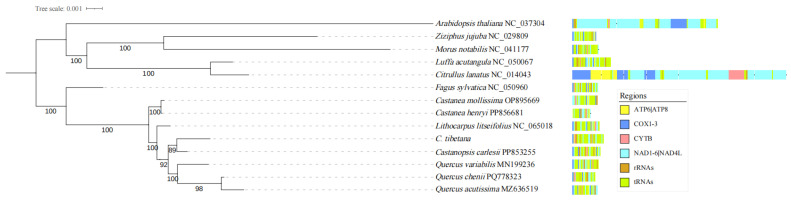
Phylogenetic tree of 13 angiosperms with 14 conserved mitochondrial PCGs.

Phylogenetic reconstruction based on 14 conserved mitochondrial PCGs from 13 angiosperm species placed *C. tibetana* within a well-supported Fagaceae clade, which was consistent with the Angiosperm Phylogeny Group classification (Figure 8). Within Fagaceae, *C. tibetana* formed a robust subclade with other *Castanopsis* species, confirming its phylogenetic position.

## 4. Discussion

Mitochondria are essential eukaryotic organelles responsible for oxidative phosphorylation and numerous other physiological processes. Plant mitogenomes exhibit striking structural complexity and exist in vivo as dynamic mixtures of linear, branched, and circular isoforms; however, they are often represented as canonical circular maps [58]. Here, we report the first complete mitogenome assembly for *C. tibetana,* offering new insights into the architecture, gene content, and evolutionary dynamics of mitogenomes within the Fagaceae family.

Among the mitogenomes of closely related Fagaceae species, the *C. tibetana* mitogenome (554,078 bp) is relatively large, as compared to *Quercus chenii* (418 kb) and *Quercus variabilis* (412 kb) [7,59]. This size variation is attributable primarily to the expansion of non-coding regions, with the accumulation of repetitive sequences serving as the main driver [60,61]. In plant mitogenomes, repetitive elements promote homologous recombination, which can generate duplications, inversions, and structural rearrangements that collectively contribute to genome expansion [62,63].

Repetitive sequences, which are ubiquitous and vary widely in abundance among plant mitogenomes [64], play a key role in shaping genome architecture. We identified 202 repeat elements, with tetrameric SSRs being the most abundant (37.1%), which is consistent with observations in other Fagaceae mitogenomes [65]. They mediate homologous recombination, generating diverse structural isomers and enabling dynamic changes in their copy number [66,67,68]. The presence of these repeats, along with their flanking regions, provides the structural basis for homologous recombination, which is known to generate alternative genomic conformations in plant mitogenomes [69]. This finding supports the prevailing view of plant mitogenomes as dynamic mixtures of multiple structural isoforms [70], a diversity well documented across Fagaceae—from the bicircular architecture in *Castanea mollissima* [71] to the tripartite circular-linear organization in *Quercus acutissima* [8] and the conventional single-circle conformation in *Fagus sylvatica* [72].

Codon usage analysis provides insights into evolutionary constraints shaping the *C. tibetana* mitogenome. Codons are fundamental units that translate genetic information into functional polypeptides [73,74]. Non-random usage of synonymous codons arises from an interplay of natural selection optimizing translational efficiency, mutation bias, and genetic drift [65,75,76]. Codon usage analysis revealed a conserved bias toward A/U-ending codons, a pattern that likely reflects a balance between mutational bias and translational selection to optimize mitochondrial tRNA abundance [77]. RSCU values quantify species-specific codon preferences: highly preferred codons (RSCU > 1) may align with mitochondrial tRNA abundance to maximize translational efficiency [77], whereas low-preference codons (RSCU < 1) exhibit species-specific patterns shaped by mutation bias or purifying selection. Rare codons may act as translational pausing sites, facilitating proper protein folding [78]. The 28 preferred codons identified here are similar to those reported in other Fagaceae species, suggesting that selective constraints on translational efficiency are highly conserved across the family. This finding implies that despite substantial structural variation, the translational machinery of Fagaceae mitogenomes remains under strong evolutionary constraints [79,80].

Compared with that of other angiosperms, the gene content of the *C. tibetana* mitogenome is highly conserved. Notably, nad7—frequently lost or transferred to the nucleus in some plant lineages [81,82]—remains intact in the *C. tibetana* mitogenome, suggesting that functional transfer has not occurred in this species. These findings provide valuable information for understanding the timing and mechanisms of mitochondrial gene transfer in Fagaceae. The 53 RNA editing sites predicted—primarily C-to-U conversions with occasional U-to-C reversions—are comparable in number to those reported in other woody plants and predominantly result in non-synonymous changes that restore conserved amino acids in respiratory chain proteins. This pattern underscores the critical role of RNA editing in compensating for genomic mutations and ensuring proper protein function [83,84,85]. In addition to RNA editing, inter-organellar gene transfer shapes mitogenome plasticity. In *C. tibetana*, we identified 20 mitochondrial plastid DNA sequences (MTPTs), totaling 6001 bp (1.08% of the mitogenome), providing evidence for ongoing inter-organellar genetic exchange [86,87]. Comparative mitogenomic analyses across Fagaceae have revealed that MTPTs are commonly present in this family, with similar frequency ranges reported in other species [8]. These transferred fragments typically represent non-functional pseudogenes, although they may occasionally contribute to functional novelty through gene capture or co-option [88].

Synteny analysis of three Fagaceae mitogenomes revealed widespread non-conserved syntenic blocks, indicative of recurrent large-scale inversions and translocations. These rearrangements demonstrate the high architectural plasticity of *Castanopsis* mitogenomes, which is likely driven by pervasive repetitive sequences [89,90] and homologous recombination [91]. Phylogenetic analysis based on 14 conserved PCGs robustly resolved the position of *C. tibetana* within *Castanopsis*, supporting the monophyly of the genus and its close relationship with *Quercus*. This topology is congruent with previous phylogenies based on chloroplast and nuclear data [9], confirming the utility of mitochondrial PCGs for evolutionary inference in this group. This high-resolution phylogeny demonstrates the utility of mitochondrial genomic data for resolving both deep and shallow evolutionary relationships within Fagaceae. Furthermore, the 202 SSRs identified represent valuable marker resources for future population genetics and conservation studies [92,93,94], enabling fine-scale assessments of genetic diversity and gene flow in this ecologically important genus.

Despite these advances, our study has several limitations. While we included *C. carlesii* and *L. litseifolius* in our comparative analyses, the absence of a broader sampling of *Castanopsis* species precludes a comprehensive understanding of mitogenome diversification within the genus. Future studies incorporating multiple species will be essential for elucidating lineage-specific structural variations and identifying genes under positive selection [13,65]. Future studies should address this gap. Additionally, functional characterization of RNA editing events and their impact on protein function will deepen the understanding of mitochondrial gene expression regulation in this ecologically important genus.

## 5. Conclusions

We report the complete mitogenome of *C. tibetana*, which is assembled as a circular molecule of 554,078 bp encoding 51 genes. Our integrative analysis revealed (i) a conserved codon usage bias across Fagaceae; (ii) a diverse repetitive landscape that provides a structural basis for recombination-mediated dynamics; (iii) 53 RNA editing sites critical for restoring protein function; and (iv) 20 MTPT sequences indicating ongoing inter-organellar gene transfer. Phylogenomic reconstruction confirmed the placement of *C. tibetana* within Fagaceae and demonstrated the utility of mitochondrial PCGs for evolutionary inference. This high-quality mitogenome provides a foundation for future research, including comparative genomics across *Castanopsis* to uncover lineage-specific adaptations and the development of cytoplasmic markers for phylogeographic and breeding studies in this ecologically and economically valuable genus. Planned comparative mitogenomic studies across multiple *Castanopsis* species will reveal lineage-specific structural variations and identify genes under positive selection, illuminating the adaptive evolution of this ecologically significant genus across diverse environmental gradients. Furthermore, the mitochondrial sequence reported here will facilitate the development of cytoplasmic molecular markers for investigating maternal inheritance patterns, phylogeography, and breeding programs in *Castanopsis* and related Fagaceae taxa.

## Data Availability

The complete mitochondrial genome assembly of *C. tibetana* generated in this study has been deposited in GenBank under the accession number PZ113713.

## Supplementary Materials

The following supporting information can be downloaded at: https://www.mdpi.com/article/10.3390/genes17040430/s1, Figure S1: Coverage depth analysis of the *C. tibetana* mitochondrial genome. Table S1: Distribution of simple sequence repeat types in the mitochondrial genomes of *C. tibetana*. Table S2: Fragments transferred from chloroplasts to mitochondria in *C. tibetana.*
