# Supplementary figures and images for: Characterization of the Complete Mitochondrial Genome of Castanopsis tibetana Hance: A Precious Timber Species
Source: Genes (Basel). 2026 Apr 7;17(4):430. doi: 10.3390/genes17040430 (PMC13116786; doi:10.3390/genes17040430)

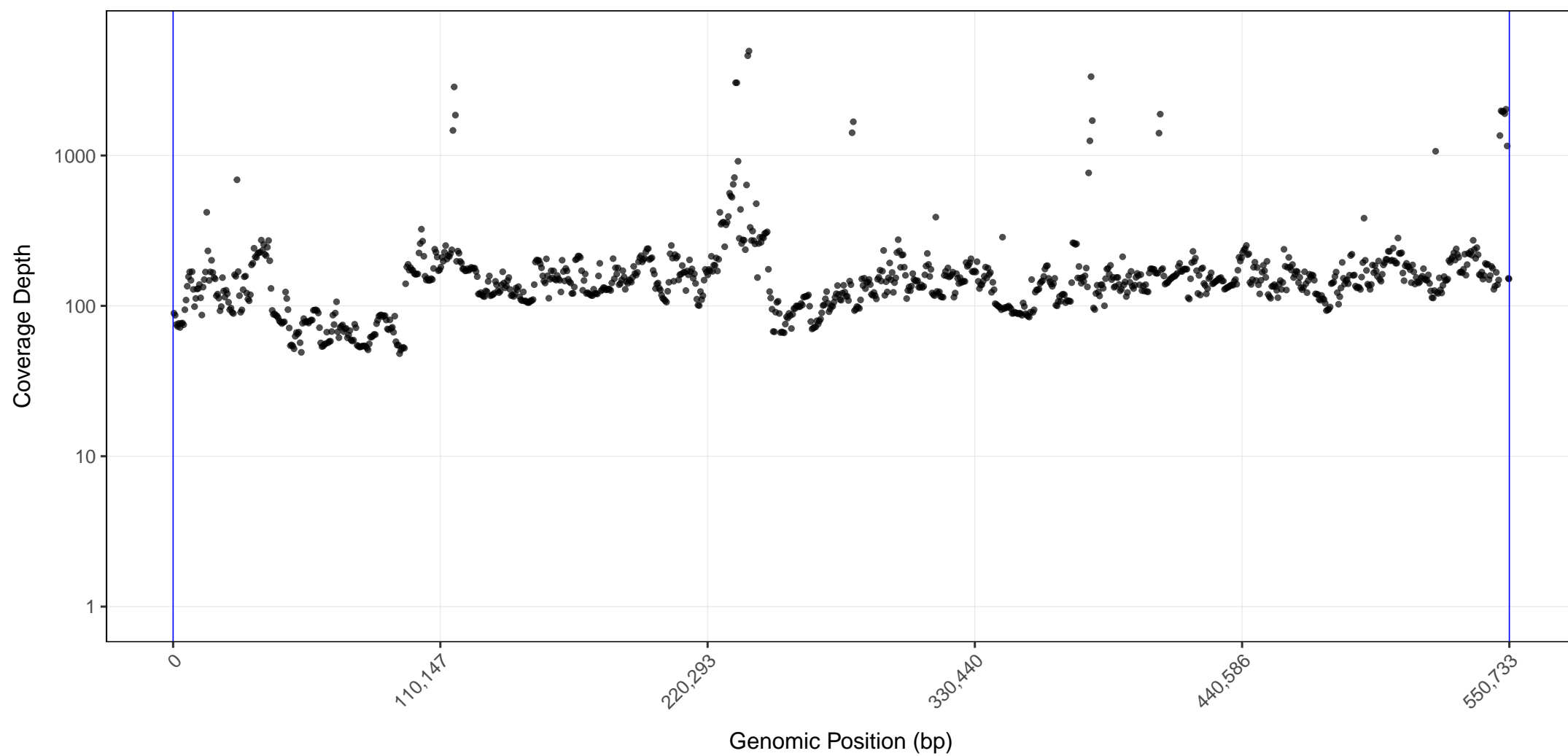

Figure S1. Coverage depth analysis of the *C. tibetana* mitochondrial genome.

Supplement: Supplementary file 1 [file genes-17-00430-s001.zip › Figure S1.pdf]
